# Young people’s experiences of vaping in their community: a co-created study between embedded researchers and local authority public health practitioners

**DOI:** 10.1177/17579139251325156

**Published:** 2025-04-28

**Authors:** L Dowrick, V Shackleton, S Nazir-Desforges, R Gettings, E Holding, M Rogerson, C Homer, C Williams

**Affiliations:** Sheffield Hallam University, Howard Street, Sheffield S1 1WB, UK; City of Doncaster Council, UK; City of Doncaster Council, UK; Sheffield Hallam University, UK; University of Sheffield, UK; City of Doncaster Council, UK; Sheffield Hallam University, UK; City of Doncaster Council, UK

**Keywords:** young people, vaping, electronic cigarettes, teenagers, co-production, embedded research, Health Determinants Research Collaboration

## Abstract

**Aim:**

This qualitative study aimed to investigate how young people understand vaping among their peers to directly inform practice within a Local Authority (LA) public health team in the North of England.

**Method:**

The study was undertaken using a co-creation model of qualitative research between LA practitioners and Health Determinants Research Collaboration (HDRC) embedded researchers. The study team collaboratively planned and collected data and analysed findings. Four focus groups were held with 17 young people aged between 13 and 23 years between May and August 2024. Participants were recruited from communities with reported high electronic cigarette (vape) use. Data analysis was informed by Braun and Clarke’s reflexive thematic analysis approach.

**Results:**

Results suggest the importance of friendship groups, social media, mental wellbeing and family in the prevalence of youth vaping, alongside the accessibility and appeal of flavours, colours, and brands of vapes. Young people identified a lack of clear information and guidance about vapes with a mixed understanding of legal frameworks. They made valuable suggestions including specialist advisors within schools, increased regulation of the accessibility and promotion of vapes, and further information about health harms.

**Conclusions:**

This study supports existing findings about the appeal of vapes to young people. The findings also add important knowledge about experiences of young people from a range of backgrounds where vaping has been identified as more prevalent locally and which mirrors health inequality data. Findings also highlight lack of awareness of legal frameworks and health harms of vaping products. Young people’s suggestions on support to reduce vaping point towards several policy and practice interventions for use in LA settings, including providing accurate and clear information on vape harms, enhancing support services and reducing vape access and appeal.

## Introduction

In recent years, young people’s (YP) electronic cigarette (vape) use in the UK has increased significantly^1^ and at a greater rate than tobacco smoking.^2–4^ Disposable vape use grew rapidly between 2021 and 2022, particularly among YP.^5,6^ Several studies have identified that many YP in the UK do not always understand the harms of vaping and nicotine.^7–9^ While less harmful than tobacco smoking, vaping is not recommended for use by non-smokers.^10^ YP’s developing lungs and brains are more sensitive to the effects of vaping and the long-term impact remains unknown.^10^ Vaping use by non-smoking YP has been identified as more prevalent among YP from disadvantaged socio-economic backgrounds and increased use has the potential to further exacerbate health inequalities.^11^ Therefore, more support targeted to YP with higher prevalence of vape use is needed for YP.

This study was undertaken as part of a newly formed Health Determinants Research Collaboration (HDRC) funded by the National Institute for Health and Care Research (NIHR) in a Local Authority (LA) in the North of England (grant reference NIHR150691). The collaboration aims to increase the LA’s capacity to develop and use research knowledge. Public health practitioners identified a need for additional information from local YP about their views and experiences of vaping to help shape effective support plans.

Existing qualitative studies have sought the views and experiences of YP in relation to vape use,^8,12–17^ but few studies have identified the kinds of support that YP would like. Studies have explored the relationship between vape use and tobacco smoking.^2,18,19^ Others have identified the role of flavours and colours as appealing to YP.^8,16,17,20^ Due to the evolving context of vape use, public health practitioners need ongoing research to understand YP’s views and use of vapes enabling timely, tailored support to address local need and health inequalities.

## Aim and objectives

This study aimed to (1) develop a greater understanding of YP’s vape use through exploring their views and experiences, (2) explore strategies for preventing and supporting the cessation of use by YP who have never smoked and (3) establish a set of recommendations to inform LA practice. The study addressed the following research questions:

1. How do YP from across the LA area perceive access to and use of vapes by their peers?
2. What do YP know about vapes and their impact?
3. What strategies do YP think would be most effective in supporting YP to understand the impact of the use of vapes?

## Methods

### Co-creation

The study was co-created by a collaborative research team of public health practitioners and embedded researchers in the LA. The co-creation incorporated all stages of the project. Co-creation is a term used here in line with the definition outlined by O’Donnell et al.^21^

### Participant recruitment

Participants were recruited through public health practitioners’ local contacts, including groups that support YP in areas with higher vaping prevalence, based on local knowledge and LA survey data. Prior to data collection, study team members visited each group to discuss the project and gauge interest in participation. All group members were provided with a Participant Information Sheet (PIS), for themselves and their families, and a consent form. YP under 16 years were provided with a parental/legal guardian consent form. A video version of the PIS was produced to ensure an accessible version of the study information for YP and their families. As some families spoke English as a second language, translated PIS and consent forms were provided in appropriate languages. Those choosing to take part who were under 16 years old were also talked through a simple assent form before starting the focus group, in addition to the parental/legal guardian consent. The study received ethical approval from Sheffield Hallam University reference ER58604236.

### Data collection

Focus groups were conducted in settings regularly used by the YP to provide a familiar, informal setting among peers. YP were not asked directly about their own use of vapes as this was not necessary for the aims of the study and could create a barrier to participation if a YP felt they may be identified as someone who vapes. Focus groups included a series of semi-structured workshop activities. A range of creative tools were developed to aid discussion and were designed to be neutral on the topic of vaping. Vignettes were drawn from anonymous questions posted on a reputable website for YP. A poster was produced which included the question prompts – who, what, when, why and how, with space for YP to add sticky notes in response. There was also an opportunity to produce posters or imaginary social media messages using art materials. Focus groups were audio-recorded and transcribed by the lead researcher. The methods and tools were developed in consultation with groups of local YP linked to the LA and a Public, Patient Involvement (PPI) group of adults linked to one of the academic institutions. The data collection took place between May and August 2024.

### Data analysis

Data analysis was informed by a reflexive thematic analysis (RTA) methodology.^22^ Study team members without prior knowledge of the methodology were provided with bespoke training to aid participation in the analysis stage including an introduction to qualitative research and RTA. Analysis was then undertaken collaboratively. This process was informed by a suggested methodology developed by Saunders et al., adapted to the needs of the project.^23^ Data familiarisation and initial coding were undertaken by the lead researcher and at least two other members of the study team per transcript. Facilitated group discussion of codes led to the emergence of key themes within the data which were subsequently organised according to the research questions established at the start of the study. Themes were written up and shared across the team to check for any further reflections.

## Results

Four groups working with YP (groups W, X, Y and Z) were recruited to the study. Across the groups, basic anonymous demographic data was collected. Eight participants were male, eight were female and one preferred not to say. Eight participants were from Gypsy or Irish Traveller, Roma or Other white backgrounds, six participants were from White British backgrounds and three were from Asian, Asian British or Asian Welsh backgrounds. YP were aged between 13 and 23 years old. The key findings are presented below in relation to the research questions. Quotes are from group participants unless stated otherwise.

### How do YP from across the LA area perceive access to and use of vapes by their peers?

Participants perceived secondary school age children, across the whole age range, as more likely to vape. Some participants also noted vaping among primary school age children. Many participants felt YP of all genders vape, with a small number perceiving girls were more likely to engage in vaping. It was also noted that YP who vaped were often not smokers previously. Participants reported that YP vaped throughout the day; it was mostly visible at the beginning and end of the school day and at weekends in public spaces. Local survey data indicated the majority of YP do not use vapes, but participants in this study emphasised that vaping was happening ‘everywhere’ and there was a clear sense that they considered vaping widespread among some YP. Participants discussed very visible locations as places where YP vape, such as around school, in local parks and community locations. They also emphasised more covert use of vapes by YP, such as in school toilets during the school day, behind closed doors in home bedrooms and away from family members. This suggests that while YP may choose to be visible through vaping in community spaces, they may also avoid being visible to schools and family members and therefore adopt different strategies accordingly:

*People do it more out of school, because in school you can be caught by teachers . . . but outside of school no one knows, just strangers, that’s what you do, have secret places*. (Group W)

Furthermore, all groups reported that YP who vape perceive it as ‘cool’ or a ‘trend’ and that it was a primarily social activity. Similarly, vaping was described as providing an opportunity to ‘show off’ either about vaping volume or through carrying out tricks and that sharing videos and photos with vapes was common, although it was unclear whether this was YP, adults or both.

*People keep them (empty vapes) like to show off how many vapes they have had*

*They put videos of them blowing rings and stuff as well*

*And they put videos on . . . whatever social media they have got really*. (Group X)

Thus, visibly vaping allows YP to demonstrate participation in a social trend.

Group X comprised of older YP linked the use of vapes to a desire to be seen to be more ‘grown up’ and to copy adult behaviours:

*I feel like kids now are in a race to grow up fast and . . . I want to do all these adult things now and it’s getting younger and younger*. (Group X)

This was particularly referenced in seeing social media content of older YP and adults, primarily people they knew, vaping while enjoying nightlife. This demonstrates the potential influence of local role models in promoting vaping:

*You see photos of people on nights out, you know vapes in their hand, everyone has got a vape in their hand*. (Group X)

In most of the groups, the concept of ‘peer pressure’ and vaping uptake was raised. Many participants rejected the notion that YP felt pressured by others to vape but did feel that there was a strong social component and desire to align with their friendship group:

*You look at all these loud rowdy teenagers with all these vapes and then look at these quiet kids with no vapes. It’s definitely like a group.* (YP1)

*God yeah, it’s cool to vape*. (YP2)

*I wouldn’t say peer pressure. But, if you don’t vape, you’re not cool sort of thing*. (YP1) (Group X)

This was similarly expressed in another group:

*If my friends have been doing it for like a year, then you want to be the same as them*. (Group W)

This suggests that there may be a strong social component to the prevalence of vaping and that narratives of peer pressure conceal more complex relationships with vape use, which is discussed further below.

A small number of participants identified vaping as a mood management tool, specifically drawing attention to its role in relation to feeling stressed:

*I have actually got a friend that does it to help with stress*. (Group Y)

Participants described the appeal of different colours, flavours and themes. They discussed the promotion of vapes with bright colours and lights in the advertising and the positioning of vapes within local shops:

*It’s ones that’s shine up, all like different flavours and stuff like that*. (Group X)

They also described a preference for disposable vapes with one participant explaining the appeal of their simplicity of use:

*Because they are easy, you don’t have to sit and wait for your liquids online, or you don’t have to go to the shop to get your liquids and coils . . . a disposable one you just take it out of the packet and smoke it, easy, easily accessible*. (Group X)

However, it was noted that the use of reusable vapes was also common among some YP.

Participants identified local shops as a primary location for obtaining vapes. They suggested a range of strategies are used to easily obtain vapes, including buying vapes directly from stores with inadequate age verification checks, having older friends or siblings purchase them, and instances of parents buying them for their children:

*Some shops need ID, but others just allow you to buy vapes*. (Group W)

*Some ask their parents to like buy them vapes, not all*. (Group Z)

Several YP also discussed the availability of vapes through friends or YP selling vapes to others.

### What do YP know about vapes and their impact?

Participants showed awareness of nicotine in vaping products. Across all groups, it was noted that there can be a shift from social vaping to more continuous use and that vaping can be addictive:

*People are genuinely getting snappy because they are not getting their vape*. (Group X)

*They are addicted to it so they want to do it inside or outside*. (Group W)

*They just keep doing it and doing it, it’s like oxygen or something isn’t it?* (Group Z)

It was unclear however whether participants understood the levels of nicotine content within vapes. One participant explained that they believed that YP do not understand the nicotine content:

*I know some people that are literally buying the disposable ones, and they will go through a full disposable in a day, or two days and they don’t understand the nicotine content is so high . . . It’s causing unnecessary nicotine addiction*. (Group X)

Views differed on whether vaping was less harmful than tobacco smoking. YP frequently moved between discussing vaping and discussing tobacco smoking. For some, vaping was perceived as better than smoking and a form of harm reduction:

*I feel like that’s why kids are leaning more towards the vaping side because they don’t want to smoke*. (Group X)

For others, however, they felt there may be more control in smoking tobacco and quantifying how many cigarettes are smoked:

*I would just say it’s no different from smoking. It’s no different from smoking normally. If, anything it could become worse than smoking normally*. (YP1)

*Yeah, because there is more nicotine in a vape*. (YP2)

*Exactly. You can control how many (cigarettes) you have in a daytime*. (YP1) (Group x)

This suggests a lack of clarity about the relative harms of smoking tobacco and vaping, but also a lack of knowledge about nicotine content within vapes. This is exacerbated further when discussions turned to the use of regulated and unregulated products. Levels of knowledge differed between groups, which may in part be attributable to age differences in participants. Older participants talked about unregulated vapes, recognising that there are legal limits and they described approximate number of puffs in a product. They confirmed vapes exceeding the limits were nonetheless still available and purchased:

*There are actually some vapes that are illegal, and some shopkeepers still sell them like the 6000 puff ones*. (Group Y)

Across all groups there was significant confusion about legal frameworks on vape access and use. Participants were often unaware that in England, the sale of vape products to under 18s is illegal, rather than the purchase, and they were confused about whether they were able to vape legally:

*I mean if it was illegal they wouldn’t serve them to young people at shops*. (Group Z)

### What strategies do YP think would be most effective in supporting YP to understand the impact of the use of vapes?

Participants outlined a range of strategies for offering support to YP. Drawing on their knowledge of smoking cessation support, participants described the potential for similar products to support cessation of vaping. This included vaping products without nicotine and access to stop vaping support. Alongside this were suggestions for increased regulation, including placing vapes out of view, reducing promotion and the removal of colours and flavours. However, one participant did raise concerns that too much regulation may lead to more use of unregulated products, as well as cigarettes.

Participants varied in how useful they believed health messages on potential harms would be in reducing vape use. Overall, participants indicated the importance of clarifying health impacts including on mental wellbeing:

*It’s bad for your health.*

*Addiction is bad for your mind*. (Group W)

There was also recognition that communicating health harms alone may not always be effective:

*I feel like even if you tell them (about the health risks) they will be like well yeah I don’t care, and they will still do it, but some will listen in the end*. (Group Z)

Participants suggested more accessible information on the harms of vapes through local advertising, school and other youth settings. Many also suggested increased support delivered within schools by specialist staff. Specialist staff were preferred to teachers, as they felt an in-depth understanding was necessary to address questions as they occur as inaccurate or delayed information could deter YP from seeking support. Interventions to provide information to YP also need to ensure that they are clear about the relative harms of vapes compared to smoking.

Overall, the strategies suggested by young people were primarily focused on solutions or support for individual behaviours such as the provision of information and on legislative responses, for example, the reduction in the accessibility and appeal of vapes. Additional important issues such as the impact of broader structural inequalities and the social determinants of health^24^ alongside the environmental consequences of vaping were not raised.

## Discussion

The findings from this study suggest YP perceive vaping as widespread and regular, particularly among secondary school age YP, that vapes are easily accessible and that there is limited knowledge about the health harms of regulated and unregulated products. This indicates a need for urgent attention to prevent widening health inequalities where higher prevalence is more likely to be found among disadvantaged socio-economic groups^11^ and the impact of unregulated product use and the long-term health harms of all vape products are not fully understood.

YP highlighted a combination of factors such as peer social groups, local promotion and the accessibility of appealing products as drivers of vape use. They suggested that the provision of appropriate information and support from accessible and reliable sources would be beneficial. The findings also indicate that engagement and discussion with YP about the social determinants of health on vape use is also needed to ensure YP are cognisant of the role of inequalities in vape use and its consequences and can contribute to exploring solutions.^25^

Several studies highlighted experimental use of vapes on social occasions.^8,12^ Discussions with the groups in this study instead emphasised more ubiquitous vaping by YP, suggesting differences in patterns of use by specific groups and the potential for widening health inequalities. Regular use has the potential for greater harms, including the development of nicotine addiction.^26^ Using unregulated vapes that may have a higher capacity creates further opportunity for nicotine addiction, alongside the intake of additional harmful chemicals. Participants indicated that vaping was prevalent among YP of all genders but with some suggestions that females may vape more than males, which aligns with growing research identifying increased use among females.^1,6^ There is an important need for support tailored to the varying patterns of vape use by a broad range of YP.

Narratives about peer pressure suggested a more complex relationship with vaping in social interactions as identified in prior research.^27,28^ Young people did not necessarily feel overt pressure to vape, but rather a desire to align with their social group and older peers. This would suggest that participation in vaping can be an activity which is part of wider identity formation.^29^ Further research exploring the shift between visible and covert use of vapes could be useful to generate additional insight. Given the social nature of vaping and the known impact of peers on its uptake, interventions which can harness the role of local older YP as role models in providing evidence-based peer information and support could also be beneficial.^30^

The diverse settings where YP vape present opportunities for health education on potential vaping harms. For example, where YP visibly vape in public, trained outreach and engagement staff could share information and discuss health harms of vaping. The hidden nature of vaping by some YP, both at home and school, highlights the need to educate families and provide them with accessible, relevant information to share with YP. Information about the legal framework and the harms of unregulated products through simple messaging and awareness raising is critical.

YP highlighted the role of colours and flavours in making vape products attractive, supporting the findings of several existing studies.^8,16,20^ Participants suggested the removal of colours and flavours may be effective strategies to reduce appeal and use by YP. The findings from this study identified very local routes for YP to obtain vapes, which facilitates easy access. While increased regulation such as the removal of vape products from view or removal of colours and flavours require national government regulation, there are important implications for LAs in relation to their approaches to the commercial determinants of health and management of illegal trading. It is also important to note that reduction in the accessibility of vapes needs to be considered alongside concerns for a potential increase in smoking^31^ and the complexity of the relationship between vaping and smoking.^18^

The findings here indicate that highlighting health concerns is important although alone may be insufficient. Prior research suggested that perceiving vapes as harmful might reduce their use and also noted that many users seemed unaware of their potential harms.^32^ This study found overall low understanding of health harms, and confusion related to the harms of regulated and unregulated products. Information and support which outlines the harms of both types of products are therefore required, alongside a range of other interventions.^33^

Participants indicated an interest in support services akin to smoking cessation services. The type and availability of this support to YP requires exploration. Although there are similarities, there are also important differences between vapes and tobacco products that need to be considered.^34^ This is supported by studies that also found an appetite from YP to stop vape use and a need to provide a variety of strategies and interventions that can engage YP with varying needs.^35,36^

Overall, our findings suggest several implications which will have relevance to other locations. The ban on disposable vapes,^37^ alongside other measures such as the Tobacco and Vapes Bill (2023-24), provides an important context for LAs. Implications include the following:

- Routes to actively engage with and provide support to YP from groups facing health inequalities.
- Clear information and training about the health harms of vapes and the legal framework suitable for different audiences.
- Support to YP to understand differences between regulated and unregulated vapes.
- Reducing the accessibility of vaping products to YP.
- Provision of services for YP to support vaping cessation.

### Strengths and limitations

The study is limited by the modest number of participants. Focus groups may result in limited engagement by some participants but open discussion and inclusion within the groups was encouraged. The study is strengthened by its engagement of YP reflecting diverse experiences and facing health inequalities and the involvement of practitioners in co-creating a study of direct relevance to their work and wider discussions about youth vaping.

## Conclusion

This study supports existing findings about the appeal of vapes to YP. They also highlight the importance of engaging with a wide and diverse range of young people in discussion about vapes to develop appropriate responses and support for YP with differing needs and experiences. The co-creation between practitioners and academics is an important partnership in practice-based research to develop appropriate recommendations.

Findings indicate an everyday use of vapes within participant’s communities and a need for knowledge of the legal framework for vaping alongside targeted health education. Local interventions will therefore need to provide both prevention and support. Importantly, where higher usage mirrors health inequality data, specific targeted action is required to prevent widening health inequalities. Although this study is specific to one geographical area, our findings will be of relevance to other LAs wishing to implement interventions to reduce youth vaping.

## Supplemental Material

sj-docx-1-rsh-10.1177_17579139251325156 – Supplemental material for Young people’s experiences of vaping in their community: a co-created study between embedded researchers and local authority public health practitioners Supplemental material, sj-docx-1-rsh-10.1177_17579139251325156 for Young people’s experiences of vaping in their community: a co-created study between embedded researchers and local authority public health practitioners by L Dowrick, V Shackleton, S Nazir-Desforges, R Gettings, E Holding, M Rogerson, C Homer and C Williams in Perspectives in Public Health
