# Supplementary material for: Young people’s experiences of vaping in their community: a co-created study between embedded researchers and local authority public health practitioners
Source: Perspect Public Health. 2025 Apr 28;145(2):113–9. doi: 10.1177/17579139251325156 (PMC12069821; doi:10.1177/17579139251325156)
Supplement: sj-docx-1-rsh-10.1177_17579139251325156 – Supplemental material for Young people’s experiences of vaping in their community: a co-created study between embedded researchers and local authority public health practitioners [file sj-docx-1-rsh-10.1177_17579139251325156.docx]

**Supplementary information: COREQ checklist**

Consolidated criteria for reporting qualitative studies (COREQ): 32-item checklist

Developed from:

Tong A, Sainsbury P, Craig J. Consolidated criteria for reporting qualitative research (COREQ): a 32-item checklist for interviews and focus groups. International Journal for Quality in Health Care. 2007. Volume 19, Number 6: pp. 349 – 357

| **Domain 1: research team and reflexivity** | | |
| --- | --- | --- |
| **Personal characteristics** | | |
| 1 Interviewer/facilitator | Which author(s) conducted the focus group? | LD co-led each of the focus groups with at least one other member of the research team for each group. |
| 2 Credentials | What were the researcher’s credentials | LD and CH have a PhD qualification. |
| 3 Occupation | What was their occupation at the time of the study | LD, EH, RG, CH are all embedded researchers.  VS, SN, CW, MR are all practitioners in public health. |
| 4 Gender | Was the researcher male or female? | RG is male. All other members of the team are female. |
| 5 Experience and training | What experience or training did the researcher have? | LD has prior experience in community work, is a qualified youth worker and researcher. RG has experience working in communities and is a researcher. EH, CH are experienced researchers with research experience with young people. VS, SN-D, MR, CW are all public health practitioners.  All members of the team without prior research experience were provided with guidance and training to support their involvement in the study. |
| **Relationship with participants** | | |
| 6. Relationship established | Was a relationship established prior to study commencement? | Contact was made with youth workers who organise and support the groups. Each of the groups were visited prior to the research to outline the research prior to the study data collection. |
| 7. Participant knowledge of the interviewer | What did the participants know about the researcher? E.g. personal goals, reasons for doing the research | The aims and purpose of the research were explained, and all researchers/practitioners attending the session introduced themselves and their roles prior to commencement of the study data collection. |
| 8. Interviewer characteristics | What characteristics were reported about the interviewer/facilitator? E.g. bias, assumptions, reasons and interests in the research topic | The different roles of the practitioners and the researchers were explained and are reported in the manuscript. |
| **Domain 2: Study Design** | | |
| **Theoretical framework** | | |
| 9. Methodological orientation and Theory | What methodological orientation was stated to underpin the study? E.g. grounded theory, discourse analysis, ethnography, content analysis | The research was a qualitative study to understand the views and experiences of participants. Data was analysed informed by a reflexive thematic analysis approach (Braun and Clarke 2006). |
| **Participant selection** | | |
| 10. Sampling | How were participants selected? E.g. purposive, convenience, consecutive, snowball | A variety of local groups were contacted based on identifying young people where higher rates of vape use have been identified. Groups were contacted using local practitioner knowledge about key contacts and groups in operation. |
| 11. Method of approach | How were participants approached? E.g. face-to-face, telephone, mail, email | Information was initially provided to youth workers about the study for them to ask if potential participants would be interested in taking part. If there was interest, an initial visit was made by members of the research team to give further information in person. |
| 12. Sample size | How many participants were in the study? | 17 |
| 13. Non-participation | How many people refused to participate or dropped out? Reasons? | We contacted a range of local groups to take part and four were interested in participating. From the four, there were no withdrawals to date. Not all regular attendees of the groups wished to participate in the research reasons were a lack of interest, because they were out of the age range or because they had not returned their legal guardian consent form. |
| **Setting** | | |
| 14. Setting of data collection | Where was the data collected? E.g. home, clinic, workplace | In community venues or workplace settings. |
| 15. Presence of non-participants | Was anyone else present besides the participants and researchers? | Youth workers attended the sessions alongside the members of the study team. |
| 16. Description of sample | What are the important characteristics of the sample? Demographic data, date | Participants were 8 male, 8 female and 1 prefer not to say. Ages ranged between 13 and 23 years. The majority were under 15 years. |
| **Data collection** | | |
| 17. Interview guide | Were questions, prompts, guides provided by the authors? Was it pilot tested? | A number of creative tools were developed to support data collection. These tools and the questions were developed with a Public participation group of adults and two groups of young people. |
| 18. Repeat interviews | Were repeat interviews carried out? If yes, how many? | No repeat interviews were carried out. |
| 19. Audio/visual recording | Did the research use audio or visual recording to collect the data? | The sessions were audio recorded. |
| 20. Field notes | Were field notes made during and/or after the interview or focus group? | Field notes were collected during the data collection. |
| 21. Duration | What was the duration of the interviews or focus group? | Focus groups generally lasted between 1 and 1.5 hours. |
| 22. Data saturation | Was data saturation discussed? | Data saturation was not discussed. |
| 23. Transcripts returned | Were transcripts returned to participants for comment and/or correction? | Transcripts were not returned to the young people. Comments and corrections are gathered at follow-up visits to each group. |
| **Domain 3: Analysis and findings** | | |
| 24. Number of data coders | How many data coders coded the data? | LD coded all 4 transcripts, 2 data coders coded 3 shorter transcripts and 2 data coders coded 1 long transcript. Discussion and comparison of coding took place to ensure consistency. |
| 25. Description of the coding tree | Did authors provide a description of the coding tree? | We did not provide a description of the coding tree. |
| 26. Derivation of themes | Were themes identified in advance or derived from the data? | Themes were derived from the data. |
| 27. Software | What software, if applicable, was used to manage the data? | Transcripts were managed using Microsoft word. |
| 28. Participant checking | Did participants provide feedback on the findings | Comments and corrections are gathered at follow-up visits to each group. |
| **Reporting** | | |
| 29. Quotations presented | Were participant quotations presented to illustrate the themes/findings? Was each quotation identified? Eg. Participant number | Participant quotations were presented with the group code from which they originated. |
| 30. Data and findings consistent | Was there consistency between the data presented and the findings? | We have endeavoured to report findings accurately and based on the data from the groups. |
| 31. Clarity of major themes | Were major themes clearly presented in the findings? | We have presented major themes in relation to the research questions for clarity. |
| 32. Clarity of minor themes | Is there a description of diverse cases or discussion of minor themes? | We have included instances of diversity and minor themes within our findings. |
